# Prospects and Challenges of Artificial Intelligence and Computer Science for the Future of Urology

**DOI:** 10.1007/s00345-020-03428-0

**Published:** 2020-09-10

**Authors:** Rodrigo Suarez-Ibarrola, Arkadiusz Miernik

**Affiliations:** Department of Urology, Faculty of Medicine, University of Freiburg - Medical Center, Freiburg, Germany

Artificial intelligence (AI) refers to the computational capability of machines to perform intellectual processes typical of human cognitive functions, such as learning, reasoning, and problem-solving. AI has permeated nearly all aspects of health care and it will increasingly continue to do so over the coming years. It initiated already a transformation on how medicine is practiced; revolutionizing traditional diagnostic, clinical decision-making and treatment-response assessment procedures. By providing more accurate and reliable clinical decisions, it will progressively play a central role in preventive medicine and will become an integral part of health care systems worldwide. Perhaps the most promising role for AI will be as an add-on to or augmentation of human providers resulting in improved efforts towards precision medicine [1]. The global revenue of the AI health care market is increasing at a rate of 40% and is expected to reach $6.6 billion by 2021 reducing treatment costs by 50% [2]. Undoubtedly, Urology is entering a new and exciting era with AI at its side; how we set about facing its new opportunities and challenges for a widespread implementation will likely have far-reaching consequences for the future of medical practice.

In this *World Journal of Urology* special issue, we gathered a series of articles from leading experts in emerging topics on the field of computer-guided technologies and AI in Urology. The aim was to present engaging and debate-generating articles that represent the current standpoint of several technologies and also point to future perspectives. Specific subfields covered by this issue include machine learning, deep learning, convolutional neural networks (CNN), radiomics, language processing, big data analysis, human–computer interaction, AI decision-making, intelligent robotics, image processing and segmentation, and smartphone-based applications.

A comprehensive, though detailed, panorama of machine and deep learning applications in urolithiasis, renal cell carcinoma, bladder and prostate cancer (PCa), outlines how these algorithms may augment surgical outcome prediction accuracy and enhance individualized medicine [3]. For instance, image texture feature extraction or radiomics has shown to be beneficial to differentiate between benign and malignant small renal masses, predict Fuhrman nuclear grade, and determine gene expression-based molecular signatures [4]. Furthermore, the emergence of AI-assisted endoscopy has prompted researchers to train CNNs with large image and video datasets to improve cystoscopic lesion detection, diagnose carcinoma in situ more accurately, and achieve diagnoses with high sensitivity and specificity [5]. In PCa, patients often face the challenge of deciding among multiple initial treatment modalities. Yu et al. evaluated IBM’s Watson for Oncology, an AI clinical decision-support system that assists uro-oncologists with evidence-based treatment recommendations. These authors found a high concordance rate with PCa patients [6]. Koo et al. utilized an artificial neural network (ANN) to predict survival outcomes according to the initial treatment and found that ANN models provided higher predictive power for 5- and 10-year progression to castration resistance PCa-free survival, cancer-specific survival and overall survival compared to traditional regression models, and may serve as online decision-making support systems [7]. Tokas et al. have used the ANNA/C-TRUS system, which utilizes PCa specific biometric signal information derived from radical prostatectomy specimens, to identify suspicious areas as biopsy targets in patients with previous negative biopsy results [8]. These targets are lesions identified by AI-trained algorithms that are not visible to the naked eye and were able to monitor image changes over a 12-year follow-up, sparing 50–75% of the usually performed biopsy cores.

The evolution of digitalization, big data analysis and AI has propelled surgical safety into a new era [9, 10]. The implementation of AI technologies in robotic surgery may be used to strengthen surgical skills acquisition feedback, surgical efficiency and guidance, and predict postoperative outcomes. Tension-sensors on the robotic arms and the integration of augmented reality methods can help enhance the surgical experience and monitor organ movements [11]. Moreover, with the advent of novel data-transmission technologies, telepresence will become a critical educational methodology profoundly impacting global health care systems [12]. The increasing incorporation of AI software into the clinical and surgical scenario will help establish telehealth concepts such as tele-training, tele-mentoring, tele-assistance and tele-surgery as an everyday reality [13].

Despite entering the era of robotic precision surgery, an unfulfilled need remains to be addressed for optimal surgical planning, navigation and training in most genitourinary diseases. Three-dimensional virtual and printed technologies have become standard practice in some centers, particularly for the management of urological tumors. These 3D models can assist in the education and training of less experienced surgeons and in patient counseling prior to the intervention [14]. The integration of 3D models with robotic platforms opens up the possibility to perform mixed reality surgeries, thereby increasing surgeons´ familiarity and confidence with the pathology and tailoring procedures to patients´ needs [15].

The evolution of smartphone technology and health-related mobile applications in medicine has radically altered health care for both patients and practitioners. Specifically in urology, intelligent software has been developed to aid in the management of urolithiasis, PCa, pediatric urology, female urology as well as for physician educational purposes [16, 17]. Although some are still in their infancy and have not demonstrated an improvement in practice thus far, others have proven beneficial over the current standard of care. For instance, PCa-related mobile phone applications have shown to be useful in physician/patient education, clinician-patient communication, shared decision-making, and biochemical recurrence risk calculation [18–21]. Altogether these efforts contribute to a favorable patient experience and increased satisfaction while also serving as a driving force for future stratified individualized care.

In conclusion, although AI and computer science will continue to provide and enhance personalized medicine, concerns regarding data protection, regulatory approvals, trustworthiness in computer diagnoses, and programming biases should be addressed in a timely fashion to ensure that these technologies function as planned. As clinicians, it is our responsibility to provide algorithms with high-quality and responsible data that will guarantee universal applicability. Ultimately, only human intuition, experience and good judgment will determine that these systems will work for the benefit of patients and health care providers.
